# Mid-upper arm circumference z-score tapes for childhood malnutrition screening: an implementation study from rural Kenya

**DOI:** 10.1186/s12889-026-28857-1

**Published:** 2026-08-12

**Authors:** Dominic Bassah, Emilie McClintic, Amy R. Sharn, Suela Sulo, Erick Agure, Grace Wothaya Kihagi, Erick M. O. Muok, Oscar Kambona Ayoma, Ina Danquah, Raissa Sorgho

**Affiliations:** 1 Center for Wellness and Nutrition, Public Health Institute, Sacramento, CA USA; 2 Global Medical Affairs and Research, Abbott Nutrition, Columbus, OH USA; 3 Global Medical Affairs and Research, Abbott Nutrition, Chicago, IL USA; 4 Transdisciplinary Research Area “Technology and Innovation for Sustainable Futures” and Center for Development Research (ZEF), University of Bonn, Bonn, Germany; 5 Heidelberg Institute of Global Health (HIGH), Medical Faculty and University Hospital, University of Heidelberg, Heidelberg, Germany; 6 Kenya Medical Research Center (KEMRI), Kisumu, Kenya; 7 Department of Health and Sanitation, County Government of Siaya, Siaya, Kenya

**Keywords:** Nutritional risk status, Mid-upper arm circumference, Mid-upper arm circumference z-score, Children, Community health volunteers, Public health nutrition, Penetration and adoption

## Abstract

**Background:**

In many remote and low-resource regions, standard methods for assessing the nutritional status of children are hindered by inadequate access to equipment, inaccurate equipment, high costs of screening, and under-resourced health facilities for malnutrition risk screening. Mid-upper arm circumference (MUAC) is a relatively simple, inexpensive, and established method for malnutrition risk screening in children, but monitoring of nutritional risk status over time can be challenging. MUAC tapes that instantly display age-specific MUAC z-scores in addition to absolute MUAC have become available. However, there are limited studies exploring the penetration and adoption of MUAC z-score tapes’ use and implementation by community health volunteers (CHVs) in community settings.

**Methods:**

As part of the ALIMUS cluster-randomized controlled trial, 15 CHVs were trained on the use of MUAC z-score tapes. The CHVs then applied the tapes during routine home visits to screen for malnutrition risk among children < 5 years of age living in Siaya County, Kenya. The study comprised 4 screenings over 1 year at 2–3 month intervals. The proportions of executed MUAC z-score screenings and nutritional risk status at each home visit were determined.

**Results:**

Of the 575 children (mean age, 15 months; SD, 5.08 months at enrollment) from 575 households, at least 85% (488/575) were screened using the MUAC z-score tape during each of the 4 screening periods. The overall proportion of malnutrition risk (overnutrition and undernutrition) declined from 18.2% in the first round to 14.1% in the fourth round of screening, increasing the proportion with normal nutritional status from 82% to 86%. Among the 451 children who completed all four screenings, 12% improved their nutritional risk status, while 7% experienced a deterioration in nutritional risk status.

**Conclusions:**

MUAC z-score tapes were repeatedly applied for nutrition risk screening in the ALIMUS study, supporting the penetration and adoption of their use by CHVs working in remote and low-resource community settings.

**Trial registration:**

German Clinical Trials Register (DRKS) DRKS00019076. Registered on 27 July 2021.

## Background

Despite progress in recent decades, childhood malnutrition and its consequences continue to threaten the ability of children to grow and thrive, especially in low- and middle-income countries. In all its forms, including undernutrition and overweight/obesity, childhood malnutrition remains a major global public health challenge. As of 2024, 150 million children under 5 years of age (23.2%) are affected by stunting, with chronic protein-energy deficiency being a major cause [1]. About 43 million (6.6%) have wasting – a form of acute undernutrition. About 36 million (5.5%) are living with overweight, and about 37 million (5.6%) with obesity [1]. Approximately 30–40% of these children live in Africa [1]. Furthermore, progress toward eliminating malnutrition has been slower in Africa than in other world regions, leaving Africa’s children at continued high risk of these preventable maladies, which have life-long adverse effects on physical and mental development, productivity, risk of infectious and chronic diseases, and mortality [2–5].

Healthy child nutrition requires addressing the direct and underlying causes of maternal and childhood malnutrition [6]. The direct causes include dietary intake and diseases; underlying causes comprise household food security, care and feeding practices, environment, and health services. Basic causes of malnutrition include resources, capital and contextual factors, along with nutrition and health policies [6, 7]. The World Health Organization (WHO) policy recommends programs to improve the identification and measurement of children at risk of malnutrition as part of efforts to prevent stunting, wasting, and overweight/obesity [8]. Age- and sex-specific anthropometric z-scores that compare to the Child Growth Standards from the WHO Multicenter Growth Reference Study are considered the Gold Standards for clinical and population-based settings [9–11]. Yet, these measures are difficult to obtain in remote and resource-constrained settings, where many community health volunteers (CHVs) and health care providers may not have accurate instruments or may not have been adequately trained in their use [12, 13]. According to WHO guidelines, admission of young children (6–59 months) into treatment programs for severe acute undernutrition is based on either weight-for-height/length of z-score <−3 or a mid-upper arm circumference (MUAC) < 115 mm. MUAC measurements are simple and low-cost, partially explaining why several treatment programs for severe undernutrition have employed a MUAC-only strategy [14, 15]. Several studies have supported the use of MUAC tapes by caregivers and CHVs for identifying non-severe childhood undernutrition in the context of community-based nutrition programs [16, 17]. However, there is no evidence-based guidance on the use of MUAC in these settings, and more research is needed [17].

**Fig. 1:**
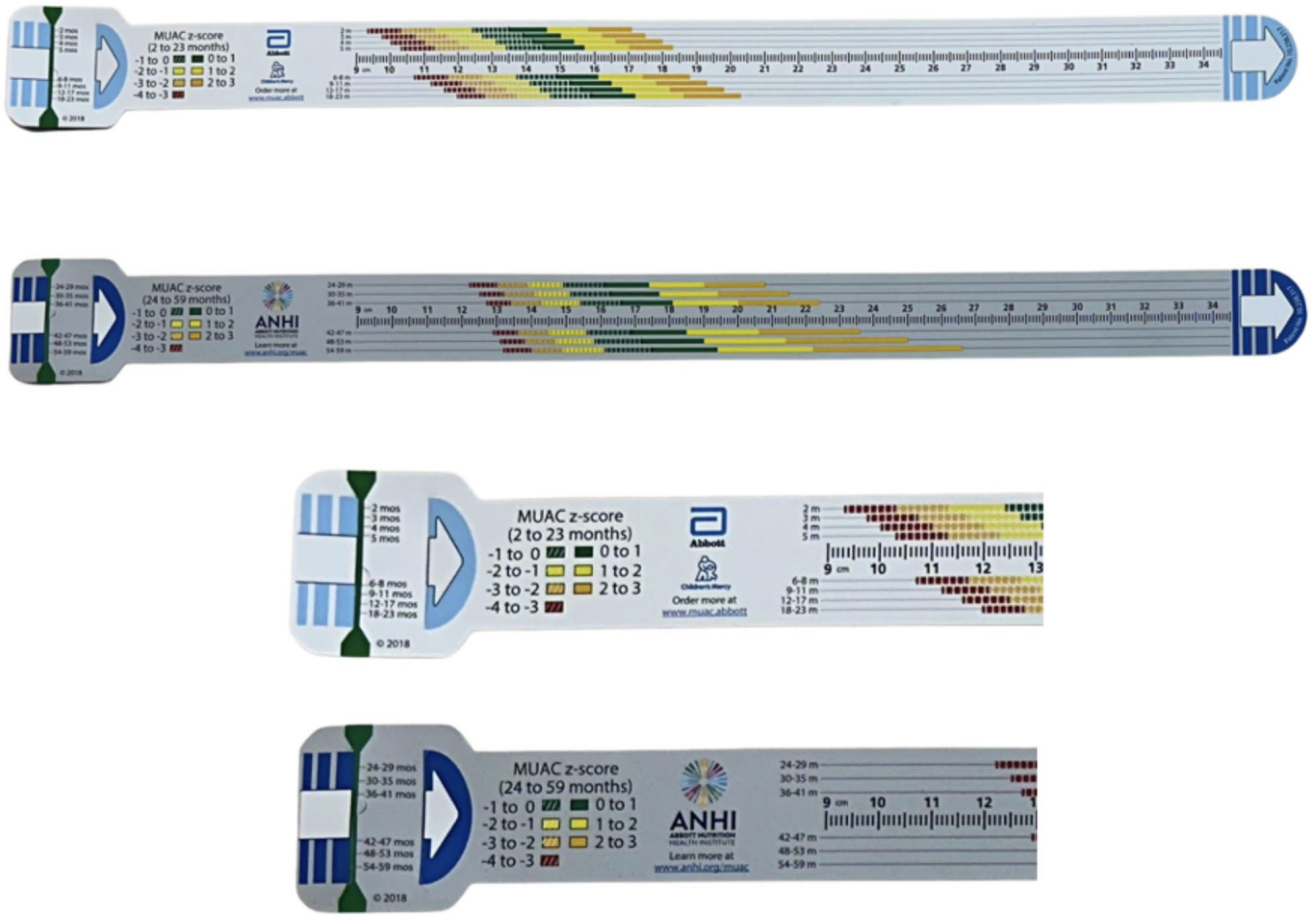
MUAC z-score tape front (white side) and back (grey side) showing the full tape (top two images) and a zoomed in version of the tape (bottom 2 images) displaying the age ranges and color codes

MUAC thresholds for malnutrition vary with the child’s age and sex [15, 18, 19] There have been efforts to develop and use MUAC scales that account for child growth (i.e., MUAC z-scores) for defining malnutrition risk status among children [15, 20–22]. MUAC z-score is recommended as one of the indicators for screening for undernutrition in the multi-ethnic population of U.S. children [23]. To facilitate the measurement and interpretation of MUAC z-scores without the use of growth charts in non-severe situations and community settings, Thaete et al. developed a MUAC z-score tape (Fig. 1). This tape allows a trained user to directly measure a child’s age-specific MUAC z-score and nutritional risk status without having to refer to growth charts and tables [24] MUAC z-scores and nutritional risk status categories shown on the tapes are based on updated and validated pediatric MUAC growth curves incorporating age-specific data. The tape indicates both over- and undernutrition. It has been validated and used to screen for the nutritional risk status of children in several settings [22, 25]. These properties turn the MUAC z-score tape into an ideal instrument for community-level monitoring of child nutritional risk status. Still, for children less than 5 years of age, there is limited information regarding the penetration and adoption of the MUAC z-score tape by CHVs and its use in community settings.

The ALIMUS project (Latin: We are feeding!) is a randomized controlled trial [26], recognized by *Nature Medicine *as a clinical trial that will change medicine in 2025, aimed to evaluate the effects of nutrition counseling and home gardening on child nutrition [27]. As part of the trial, the MUAC z-score tape was used for nutrition risk screening among households in southwestern Kenya by CHVs. The main goal of this study was to determine the adoption (early uptake) and penetration (continued use) of using the MUAC z-score tape for community-level nutrition risk screening and monitoring. The specific objectives were to (i) determine the proportions of screened children at each of four home visits, (ii) identify child malnutrition risk status at each visit, and (iii) uncover improved nutritional risk status from the first to the fourth visit.

## Methods

### Study design and setting

This longitudinal implementation research study was conducted with the Kenya Medical Research Institute in rural Kenya. The background, rationale, design, and protocol of the ALIMUS trial have been published elsewhere [26]. In brief, the ALIMUS trial is a cluster-randomized controlled trial (cRCT) in rural Kenya that started in September 2021. It enrolled 300 households in the intervention group and 300 households in the control group. The population in Siaya County within the Health and Demographic Surveillance System area is mainly small-scale farmers - agriculture and fishing, with a typical diet rich in starchy staples and supplemented with few legumes. According to the 2022 Kenya Demographic and Health Survey, the prevalence of under-five stunting in Siaya County is 19% compared with the national prevalence of 18% [28].

### Study population and enrollment

Study participants were children, aged 6–23 months at enrollment into the ALIMUS cRCT. Before participants were enrolled into the ALIMUS trial, caregivers were informed about the study in their preferred language of English, Swahili, or Luo, and afterwards provided informed, written consent. Additional recruitment and enrollment information is outlined in the published ALIMUS trial protocol [26].

### The ALIMUS intervention

This longitudinal implementation research study was embedded within the ALIMUS trial, which started in July 2021 and spanned 2 years of implementation from January 2022 until December 2024. The intervention consisted of individualized nutrition counseling and home garden implementation [26]. Nutrition counseling comprised one group counseling session per household, in groups of 20 households, and individual counseling sessions every 2–3 months during the 2-year intervention period. Home garden implementation comprised setting up home gardens, providing seeds, inputs, and conducting various training sessions. Home gardening lesson topics included: organic pesticide production; composting; nursery establishment; land preparation; home garden construction; planting, weeding and plant protection; harvesting, preservation and storage; and garden maintenance. Participants in the control arm participated in one group counselling session while those in the intervention arm received individual counselling every 2–3 months.

### Malnutrition risk screening by MUAC z-score tape

Before data collection, 15 CHVs were trained by the researchers on the use of MUAC z-score tapes as well as the interpretation and documentation of the results.

The Siaya County Nutrition Coordinator selected CHVs in the ALIMUS trial to participate in the MUACz component of the study. All 15 CHVs involved in the ALIMUS trial volunteered to attend the MUACz training and implemented the MUACz screening program. Inclusion criteria comprised working in Siaya County, being a participant of the ALIMUS trial and participating in the MUACz tape training. Twenty hours of in-person training in groups were conducted for CHVs over the course of three days, employing presentations, videos, and hands-on practice of anthropometric measurements using the MUACz tape. The MUACz training introduced the tape to the attendees, emphasizing its distinct features and sensitivity to age-specific malnutrition. The training equipped CHVs with the skills and hands-on experience to conduct nutrition screening using the MUACz tape. Successful training completion was marked by attendance at all training sessions, completing all case studies and practical screening assignments. Researchers assessed screening competency as part of the training. Each CHV was assigned a case study to assess the correct screening and classification of malnutrition risk. Researchers observed each CHV perform screening and recording during the piloting phase of the project.

CHVs also participated in regular reminders and debriefing sessions to maintain high-quality data collection. CHVs were instructed on how to locate the middle of the less-active arm and to take two consecutive MUAC z-score readings of the same arm, using the age-specific markings on the z-score tape corresponding to the child’s age. The mean of the two separate readings was recorded. When the MUAC z-score readings were more then 0.8 cm apart, a third reading was conducted. The mean of the two closest readings was recorded as the final measurement. CHVs were assigned to households in communities where they conducted their routine nutrition and health assessment, due to the nature of the ALIMUS intervention CHVs could not be blinded to the households assigned study arm.

**Fig. 2:**
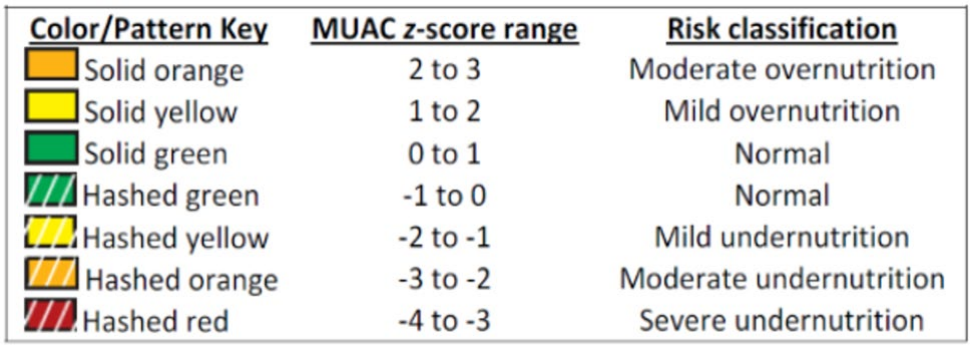
Table showing color coding on MUAC z-score tape, their corresponding z-score ranges and risk classification

Using the child’s measured arm circumference and age, a corresponding z-score range was identified and recorded from the color-coded and shaded tape (Figs. 1 and 2). Malnutrition risk was indicated by both the MUAC z-score and the tape’s color/shading pattern (hashed vs. solid). Z-scores from + 1 to 0 to − 1 fall within the normal range (hashed and solid green). Undernutrition was classified as mild (− 1 to − 2, hashed yellow), moderate (− 2 to − 3, hashed orange), and severe (− 3 to − 4, hashed red). Overnutrition was classified as (1 to 2, solid yellow) and moderate (2 to 3, solid orange), following guidelines from the Academy of Nutrition and Dietetics and the American Society for Parenteral and Enteral Nutrition [23] (Fig. 2).

Between April 2023 and September 2024 during the ongoing ALIMUS trial, CHVs visited each participating household in the intervention group and in the control group four times during the 1-year study period. All CHVs were instructed to conduct 4 screenings over the course of 1 year in each of their assigned households with an estimated 2–3-month intervals. The date of screening for each household depended on the individual CHV’s schedule, household availability and presence of the participating child in the household for screening. During the household visits by a CHV, MUAC z-score screenings were performed on participating child using MUAC z-score tapes (licensed by Abbott Laboratories, Chicago, IL, USA) from The Children’s Mercy Hospital (Kansas City, MO, USA). Children with screening results indicating severe acute undernutrition by MUAC z-score <−3 (red hashed) were referred to a healthcare provider for further evaluation, diagnosis, and treatment. MUAC readings (in cm and z-scores) were recorded on smartphones into the secure data entry platform Survey Solutions (version 25.4, World Bank Group, Washington, D.C., USA) and later uploaded to a password-protected secure server at Heidelberg University Hospital, Heidelberg, Germany.

### Data analysis

The proportion of children who participated in MUAC z-score screenings at each visit in the intervention and control groups were compared by Chi-square tests. Demographic characteristics of 575 children are presented descriptively at each visit, including means and standard deviations (SD) for age in months and frequencies (%) and absolute counts for sex.

For analysis of the malnutrition risk status, age-specific MUAC z-scores were categorized into one of six groups signaled by color coding and shading (hashed and solid) on the tape. Figure 2shows the respective cut-offs and their interpretations as laid out by the Academy of Nutrition and Dietetics and the American Society for Parenteral and Enteral Nutrition [23]. The proportions of children with normal, mild, moderate, and severe malnutrition risk status are displayed for each timepoint (4 times within 1 year) in absolute counts and frequencies (%).

Additional subgroup analyses were conducted to explore improvement in malnutrition risk status over the four timepoints. We calculated the differences in malnutrition risk at each timepoint between the study groups. In the calculation of malnutrition risk at each timepoint, only complete cases were used. Participants with missing data at a particular home visit were dropped from the analysis of risk status at that time point and included at timepoints where data were available. We conducted a sensitivity analysis to assess the robustness of our findings. Children who shared the same household (cluster) were excluded from this analysis. For subgroup analyses, MUAC z-scores were categorized into three groups: undernutrition (MUAC z-score <−1), overnutrition (MUAC z-score > 1), or not at risk (−1 ≥ MUAC z-score ≤ 1) and compared by Chi-square tests. A p-value of < 0.05 was considered statistically significant. All analyses were performed using R Studio (version 4.3.1).

## Results

### Demographic characteristics

A total of 575 children from 575 households across 169 villages in Siaya County, Kenya, were enrolled. At enrollment in the ALIMUS trial, the mean age of participants was 15 months (SD = 5.08), and 54% were male (*n* = 312). Mean age and sex distribution across the four screening rounds are summarized in Table 1.

**Table 1:** Demographic characteristics among ALIMUS trial and across four rounds of MUAC z-score screenings, *N* = 575

Visit	*N*	Mean age in months (SD)	Male, *n* (%)
At ALIMUS trial Enrollment	575	15 (5.08)	312 (54.3)
MUAC z-score Screenings			
First	499	34 (5.32)	269 (53.9)
Second	503	36 (5.23)	271 (53.9)
Third	493	39 (5.51)	265 (53.8)
Fourth	488	41 (5.90)	259 (53.1)

### Participation in MUAC z-score screenings

The number and percentage of participants screened using MUAC z-score tape during the 4 screening periods is shown in Table 2. Of the 575 participants, 78% (451/575) completed all four screening rounds, and 89% (511/575) completed at least two rounds. The proportion of children participating in MUAC z-score screening at each visit was at least 85% (488/575) and remained stable (*p* = 0.59). Of the 499 children who initially participated in the first screening round, 98% (488/499) completed the fourth round of screening. Overall, 9% (50/575) of children missed all four screenings and 22% (124/575) missed at least one screening. Missed screenings were largely due to parents/caregivers declining MUAC z-score screening or the child’s absence at the time of the visit. The baseline characteristics of the children who were not screened did not differ from those of children who were screened.

**Table 2:** Screening proportions and malnutrition risk status of participants at each of 4 screening rounds, *N* = 575

		Screening Round			
		First	Second	Third	Fourth
Total Screened, n (% of 575)		499 (87)	503 (88)	493 (86)	488 (85)
z-score	Malnutrition Risk Status, n (% of round total)				
≤−1 or ≥ 1	Malnutrition, All Forms	91 (18)	57 (11)	79 (16)	69 (14)
<−1	Undernutrition, Mild to Severe	67 (13)	29 (6)	36 (7)	32 (7)
> 1	Overnutrition, Mild to Moderate	24 (5)	29 (6)	43 (9)	37 (8)
−4 to − 3	Severe Undernutrition	1 (0.2)	2 (0.4)	2 (0.4)	0 (0)
−3 to − 2	Moderate Undernutrition	3 (0.6)	1 (0.18)	2 (0.4)	1 (0.2)
−2 to − 1	Mild Undernutrition	63 (13)	25 (5)	32 (7)	31 (7)
−1 to 1	Normal/Not at Risk	408 (82)	446 (89)	414 (84)	419 (86)
1 to 2	Mild Overnutrition	22 (4)	28 (6)	41 (8)	37 (8)
2 to 3	Moderate Overnutrition	2 (0.4)	1 (0.2)	2 (0.4)	0 (0)

### Improving nutritional risk status

At the first screening using MUAC z-score tape,18% (91/499) of children had one form of malnutrition risk. Overall, the frequency of malnutrition risk declined throughout the study period across all four time points, with 11.3% (57/503) at the second round, 160% (79/493) at the third round, and 14.1% (69/488) at the fourth round (*p* = 0.017). Furthermore, the Cochran-Armitage test showed no statistically significant progressive reduction in the proportion of malnutrition across the four time points (*p* = 0.288).

Of those children who completed all four screening rounds (n = 451), 12% (52/451) improved their malnutrition risk status, and 7% (32/451) experienced a worsening of their malnutrition risk status between the first and the fourth home visits. A McNemar test showed a significant change in nutritional risk status between the first and fourth screening rounds (p < 0.001). A change from over/undernutrition to “not at risk’ was considered an improvement in risk status, while a change from “not at risk’ to over/undernutrition was considered a worsened risk status. Table 3 shows the malnutrition risk status of children between the first and fourth screenings for children who completed all four rounds of screening.

**Table 3:** Malnutrition risk status between the first and fourth rounds of screening

First Round	Fourth Round	
	Not at Risk	Over/undernutrition
Not at Risk	334	32
Over/undernutrition	52	33

### Differences in malnutrition risk status between intervention groups over time

Table 4 presents the frequency of malnutrition risk by study group across the four screening rounds. There was no statistical difference in the proportion of screening across the four screening rounds between the control group and the intervention group. Across the four rounds of screening, a significant decrease in the proportion of undernutrition was observed among children in both the control arm and intervention arm (*p* = 0.005 and 0.003, respectively). The Cochran-Armitage test confirmed a linear decrease in the proportion of malnutrition in both arms across the four time points (*p* = 0.016 & *p* = 0.014 in the intervention and control group, respectively). The proportion of children with overnutrition did not change significantly from the first to the fourth round of screening in both study groups.

**Table 4:** Malnutrition risk by intervention group across four screening rounds, *N* = 575

	Screening Round				
	First	Second	Third	Fourth	*p*-value*
Participants Screened, n (%)	499(87)	503(88)	493(86)	488(85)	0.59
Control	226	229	217	216	0.407
Intervention	273	274	276	272	0.944
Undernutrition (MUAC z-score <−1), n (%)					
Control	30 (13)	12 (5)	18 (8)	12 (6)	0.005
Intervention	37 (14)	16 (6)	18 (7)	20 (7)	0.003
Overnutrition (MUAC z-score > 1), n (%)					
Control	8 (4)	12(5)	16(7)	18 (8)	0.145
Intervention	16 (6)	17(6)	27(10)	19 (7)	0.271

* Chi-square test

## Discussion

In this implementation research project of 575 young children, the MUAC z-score tape showed substantial adoption in community-level nutrition screening, with at least 85% of participants screened at each of the four home visits. Over this period, malnutrition risk declined from 18% to 14%, with similar reductions observed in both the control and intervention groups. Among children completing all four screening rounds, 12% improved their malnutrition risk status.

### MUAC z-score tape can be adopted as a screening tool in community settings

Commonly known as uptake, adoption is the intention, initial decision or action to implement an innovation. It can be measured from the perspective of organizations or providers [29]. The initial uptake of the MUAC z-score tape for malnutrition screening reached 88%, indicating a large majority of households adopted and accepted the tape for malnutrition screening*.*

The high proportion (≥ 85%) of MUAC z-score screening throughout the study period suggests that MUAC z-score tapes can reach constant penetration for malnutrition risk screening in community settings. Penetration refers to the degree to which a practice is integrated into a service setting and its subsystems [29]. These findings are consistent with other community-based MUAC z-score tape screening studies among children over five years of age [22, 25]. The user-friendliness of the tapes may be a contributing factor to the adoption and penetration, as many dietitians who have used the tapes have found it easy to use, efficient, convenient and among the anthropometric tools, a preferred alternative [24]. Thorough training of CHVs, strong rapport with caregivers, and the similarity of the MUAC z-score tape to the well-established MUAC tape may have influenced screening uptake, as shown by the findings from a separate qualitative publication in the same study population, where CHVs reported positive experiences using the MUAC z-score tape for nutrition screening [30]. In resource-restricted areas, where routine nutritional status assessments may be lacking, the MUAC z-score tape can be a viable, low-cost and low-burden alternative for malnutrition risk identification following thorough training [22] and integration into routine home visits. Given that 78% of children enrolled in the study completed four rounds of screening, the tape has the potential to serve as a tool for continuous household screening of children’s malnutrition risk status, thereby reducing the need for multiple visits to a healthcare facility. The results of our study support the applicability of MUAC z-score tape use by trained CHVs to screen the malnutrition risk status of young children.

### Improvements in nutritional status with regular screening over time

Throughout the four home visits and screenings, the proportion of children with malnutrition decreased from 18% to 14%. While the proportion of undernutrition (mild to severe) decreased from 13% to 7%, the proportion of overnutrition (mild to moderate) increased from 5% to 8%. The observed reduction in the proportion of malnutrition risk over time has been seen in other studies that employed CHV service-home visits, referrals, counselling, and follow-up [31] and thus, suggests a similar association in the present study population. It is also important to consider that regular screening and sustained CHV engagement with caregivers may have increased awareness of child nutritional status and supported appropriate feeding practices, potentially contributing to the observed changes during the study period [32].

An increase in the proportion of children with overnutrition suggests that additional education is needed on the nuances of nutrition. Children recovering from undernutrition are often at risk of developing overweight or obesity if not provided with adequate nutrients in appropriate proportions to support healthy child growth [2]. The MUAC z-score tape’s ability to identify a broad spectrum of nutritional risk beyond undernutrition, suggesting that traditional MUAC screening approaches could have underestimated the true burden of compromised nutritional status. Such an immediate and understandable interpretation from the MUAC z-score screening results [30] can serve as a reminder for caregivers and community stakeholders about child nutritional risk status, especially when directly paired with a nutrition counseling session on the importance of appropriate nutrition for child growth and development across different presentations of malnutrition. The ease of use of the MUAC z-score tape warrants further investigation, although the ability of CHVs to screen for malnutrition risk as part of this study supports their use [31]. Furthermore, future research and intervention initiatives should investigate the scaling out [33] and up of the MUAC z-score tape screening in communities and its integration into existing healthcare routines.

### Strengths and limitations

Our study used a participatory approach to implement the screening program with oversight by the MOH and was implemented by CHVs who have an in-depth understanding and experience of the healthcare landscape in the communities. The study also demonstrated the participation and retention rates of malnutrition risk screening with the novel MUAC z-score tape in communities where food and healthcare resources may be limited. Although screening is a first step in detecting malnutrition, the importance of a health care system that can further assess, diagnose, and treat malnutrition remains imperative. This study showed that CHVs could be successfully trained to use a MUAC z-score tape as a screening tool to identify children who may be at risk for malnutrition presenting as both under- and overnutrition. Once children with malnutrition risk were identified, the facilitators trained by the ALIMUS study had the skills to begin conversations with caregivers about the importance of nutrition in childhood for healthy growth trajectories. They were also able to encourage follow-up with a healthcare professional for further evaluation and treatment if indicated. Community stakeholders and caregivers are thus alerted to the importance of taking action to improve access to food and proper nutrition.

However, this study also had limitations. This study helped to guide the usability of the MUAC z-score tape but given the participant eligibility criteria (families with access to water and land, living in the perimeters of weather stations) of the ALIMUS trial, the results cannot be extrapolated or generalized to all children. In our study, the MUAC z-score tape was not directly compared with the nutritional risk status determined by other methods, which would be necessary to validate the tool (e.g., weight-for-height/length z-score), as this study focused on the adoption and penetration of the new tool [29].

The high penetration and adoption could be due to the Hawthorne effect as CHVs knew they were being watched. Results may not be generalizable due to the strict inclusion criteria of access to water, land, and proximity to weather stations. Malnutrition risk may be higher in households with limited resources compared to the ALIMUS study population.

Our study did not assess variation in the use of the MUACz tapes as well as the impact of screening results on appropriate referrals and follow-up.

Alternatively, MUAC z-scores may provide a valuable second perspective on malnutrition risk by identifying malnutrition risk outside the scope of the original MUAC tape through age and sex z-scores for both undernutrition and overnutrition, especially in resource-restricted settings. Secondly, the study was conducted within the context of an established protocol, so CHVs and participant households were already comfortable with each other. CHVs had built a high level of trust within study communities, facilitating the integration of MUAC z-score screening of children in routine household visits after rigorous training. Thus, it is unclear if the results will translate to community settings that have less familiarity with community health screenings.

To account for household clustering, households with multiple children were excluded from the analysis. This approach may impact the estimates of malnutrition risks. More sophisticated methods that account for clustering while retaining all observations could provide more efficient estimates.

Additionally, only basic participant demographic information from the sample of 575 was collected, so we could not assess malnutrition risk trends among specific participant groups or establish prevalence, i.e., those with different comorbidities or diagnoses. Without such fine-tuning, we cannot rule out confounding.

## Conclusions

Our study showed high screening participation, stable penetration over time, and observed changes in malnutrition risk status. Age-specific MUAC z-score tapes can be adopted by CHVs and may penetrate malnutrition risk screening for young children in a low-resource community setting in Kenya after rigorous training and support to CHVs. Further research specifically on tool validation and comparison could address concordance of this method with other malnutrition screening methods, as well as the value of immediate color-coding and age-specific presentation of malnutrition risk screening results. Future work should investigate the scaling out [33] and up of the MUAC z-score tape screening in communities and its integration into existing healthcare routines outside of the ALIMUS trial.

## Data Availability

The datasets generated and/or analyzed during the current study are not publicly available to preserve participant privacy but are available from the corresponding author on reasonable request.

## Abbreviations

CGHR: Centre for Global Health Research
CHV: Community Health Volunteer
DFG: German Research Foundation
HIGH: Heidelberg Institute for Global Health
KEMRI: Kenya Medical Research Institute
MOH: Ministry of Health
MUAC: Mid-Upper Arm Circumference
RCT: Randomized Controlled Trial
SERU: Scientific and Ethics Review Unit
WFP: World Food Program
WHO: World Health Organization
ZEF: Center for Development Research
